# Thermal plasticity of adrenaline-mediated, frequency-dependent calcium homeostasis in rainbow trout ventricular cardiomyocytes

**DOI:** 10.1242/jeb.251460

**Published:** 2026-02-02

**Authors:** Ilan M. Ruhr, Gina L. J. Galli, Holly A. Shiels

**Affiliations:** ^1^ Biomedical Research and Innovation Centre, School of Science, Engineering and Environment, University of Salford, Salford M5 4NT, UK; ^2^ Division of Cardiovascular Sciences, Faculty of Biology, Medicine and Health, University of Manchester, Manchester M13 9NT, UK

**Keywords:** Sarcoplasmic reticulum, Ryanodine receptor, SERCA, Ca^2+^ transient, Acute warming, Thermal tolerance

## Abstract

The effect of global warming on rising aquatic temperatures is producing ever-steeper thermoclines. Fish encountering these sharp changes in water temperature might experience an acute-warming stress. Temperature is the most dominant environmental factor affecting heart function in fish, and without compensatory mechanisms as temperatures rise (e.g. higher heart rate), it could imperil cardiovascular performance. To enhance heart function during acute warming, fish release adrenaline to boost Ca^2+^ influx in heart cells (cardiomyocytes). However, the relationship between acute warming, elevated heart rate, adrenergic stimulation and intracellular Ca^2+^ handling is not well understood at the cellular level. In this study, we investigated the interplay between these key functional drivers in isolated ventricular cardiomyocytes of rainbow trout, at either their acclimation temperature of 10°C or following acute warming (22°C). A subset of cardiomyocytes from each group was treated with adrenaline, sarcoplasmic reticulum (SR) inhibitors (that inhibit intracellular Ca^2+^ cycling via the SR) or both, whereas pacing frequency was simultaneously increased (simulating faster heart rate). Using epifluorescent microscopy, we measured intracellular Ca^2+^ transients (Δ[Ca^2+^]_i_) and Ca^2+^-cycling kinetics. Across all pacing frequencies, we found no differences in Δ[Ca^2+^]_i_ between control (untreated) 10°C and 22°C cardiomyocytes, and that adrenaline had a positive inotropic effect at both temperatures, but was less effective at 22°C. SR inhibition had no effect on Δ[Ca^2+^]_i_, but was associated with a greater incidence of irregular Δ[Ca^2+^]_i_. Our data suggest that acute thermal stress can disrupt Ca^2+^-homeostatic mechanisms in trout cardiomyocytes, potentially disrupting whole-heart contractility as global temperatures rise.

## Teaser

**Summary:** Analysis of the relationship between acute warming, elevated heart rate, adrenaline and inhibited SR intracellular Ca^2+^ cycling shows that acute thermal stress disrupts mechanisms regulating Ca^2+^ homeostasis in trout cardiomyocytes, which might underlie disruption of whole-heart contractility.

## INTRODUCTION

Environmental temperature is the prime directive regulating energy expenditure and metabolic rate in fishes (Gillooly et al., 2001). Highly aerobic organs, such as the fish heart, are profoundly affected by temperature fluctuations (Farrell et al., 2009). The impact of acute temperature changes (measured in seconds to hours) on the fish cardiovascular system is well studied (reviewed by Eliason and Anttila, 2017; Farrell and Smith, 2017; Shiels et al., 2024; Vornanen, 2016, 2017; Vornanen and Hassinen, 2016) and has growing relevance in the context of warming aquatic ecosystems owing to climate change. Fish cardiovascular systems have thermal optima, above which function plateaus or declines, which impacts performance traits that include swimming, foraging and reproduction, ultimately affecting survival (Brett, 1971; Eliason et al., 2011; Franklin et al., 2013; Gilbert et al., 2020; Safi et al., 2019; Steinhausen et al., 2008). The mechanisms leading to cardiac collapse at high temperatures is an active area of research, yet the processes are still not fully understood. Several studies suggest that contractility is compromised at high temperatures, owing to the negative inotropic effect of high heart rates (i.e. a negative force–frequency response) and the waning effect of adrenergic stimulation. Here, we have examined the interactive effects of contraction frequency and adrenergic stimulation on rainbow trout (*Oncorhynchus mykiss*) ventricular cardiomyocyte function during acute warming, to provide mechanistic insight into cardiac thermal stress.

Fish exposed to an acute increase in temperature experience a rise in tissue metabolic demands. Higher tissue metabolism is satisfied by an elevation in cardiac output to provide higher rates of oxygen (O_2_) delivery, which is achieved by elevating heart rate (e.g. Brodeur et al., 2001; Ekström et al., 2019; Gamperl et al., 2011; Haverinen et al., 2017; Haverinen and Vornanen, 2020; Keen and Gamperl, 2012; Petersen et al., 2011; Safi et al., 2019; Sandblom and Axelsson, 2007; and reviewed by Eliason and Anttila, 2017; Vornanen, 2016, 2017). If temperature continues to rise, heart rate increases until approximately 1–2°C before the fish reaches its critical thermal maximum (CT_max_). At this point, cardiac output weakens, heart rate becomes irregular and/or ventricular contractility exhibits irregular heartbeats (arrhythmias) (Anelli et al., 2004; Badr et al., 2016; Clark et al., 2008; Gilbert et al., 2020; Gollock et al., 2006; Strowbridge et al., 2025; Vornanen et al., 2014; reviewed by Vornanen et al., 2024). Ventricular arrhythmias in fish are generally rare, but become more pronounced at critically high temperatures, where they usually present as bradycardia, first-degree heart block (slowing of conductance across the A–V node) and missing QRS complexes in heart beats (Vornanen et al., 2024). Conduction failure has been attributed to a thermally induced mismatch between the depolarising and repolarising ion currents responsible for propagating action potentials across the heart (Haverinen and Vornanen, 2020; Vornanen et al., 2014). Although adrenergic stimulation can counteract the effects of acute warming or high heart rates (Aho and Vornanen, 2001; Keen et al., 1993; Wood et al., 1979), studies using *in vivo*, *in situ*, isolated heart and isolated muscle-strip preparations show that as heart rate and/or pacing frequency quickens with acute warming (Farrell et al., 1996; Gilbert et al., 2020; Keen and Farrell, 1994; Overgaard et al., 2004; Sutcliffe et al., 2020), the force of contraction inexorably weakens (Anelli et al., 2004; Rocha et al., 2007; Shiels and Farrell, 1997; Shiels et al., 1999; Tittu and Vornanen, 2001; Vornanen, 1989). Because these effects were observed in isolated tissue, it suggests that they are intrinsic to the tissue and, possibly, individual cardiomyocytes.

Cardiomyocyte contraction and relaxation in fish occurs by excitation–contraction (EC) coupling, during which an action potential triggers the influx of Ca^2+^ from the extracellular space, which elevates cytosolic free (intracellular) [Ca^2+^] ([Ca^2+^]_i_) and triggers the release of Ca^2+^ stored in the sarcoplasmic reticulum (SR), through ryanodine receptors (RyRs) (Vornanen et al., 2002). The relative contribution of Ca^2+^ from each of these sources varies with species, tissue type and developmental stage (Shiels and Galli, 2014; Vornanen et al., 2024, 2002). Regardless of source, the rise in cytosolic Ca^2+^ is required for Ca^2+^ to successfully bind to troponin-C and engage the myofilaments, leading to cross-bridge cycling and cardiomyocyte contraction (Gillis and Tibbits, 2002). Relaxation occurs by the removal of cytosolic Ca^2+^ back across the sarcolemmal membrane, through L-type Ca^2+^ channels (producing the L-type *I*_Ca_) and/or by pumping Ca^2+^ back into the SR, via the sarco-/endoplasmic reticulum Ca^2+^-ATPase (SERCA). The change in [Ca^2+^]_i_ during EC coupling is known as the Ca^2+^ transient (Δ[Ca^2+^]_i_), and underlies the force of contraction when muscle length is constant.

Temperature, adrenaline and pacing frequency exert their influence on contractility by modulating EC-coupling pathways. Adrenaline elevates Ca^2+^ entry (*I*_Ca_) and stimulates SERCA activity (Shiels and Galli, 2014; Shiels et al., 2024). However, the cardioprotective effect of adrenaline is frequency and temperature dependent. Adrenaline is more effective at raising Δ[Ca^2+^]_i_ and, thus, strengthening contractile force at slower pacing frequencies, whereas its effects at faster pacing frequencies depend on SR Ca^2+^-cycling efficiency. This conclusion is drawn from a range of studies on fish with different life histories, in which the force–frequency relationship has been examined in isolated muscle-strip preparations [e.g. rainbow trout (Shiels and Farrell, 1997; Shiels et al., 1998), sockeye salmon (Goulding and Farrell, 2020), Pacific mackerel (Shiels and Farrell, 2000), marbled swamp eel (Rocha et al., 2007), blackfin icefish (Skov et al., 2009) and black rockcod (Skov et al., 2009)]. The sensitivity of the fish heart to adrenaline also decreases with acute warming (Galli et al., 2009; Kubly and Stecyk, 2019; Methling et al., 2012; Shiels et al., 1998, 2003). It is unclear why acute warming desensitises cardiac tissue to adrenaline, but it might explain the occurrence of arrhythmias, as cells struggle to remove cytosolic Ca^2+^ during diastole, possibly because of impaired intracellular Ca^2+^ handling, the development of Ca^2+^ overload and the concomitant faster heart rates.

Given the interplay between pacing frequency, temperature and adrenergic stimulation at the tissue level, our overall aim was to examine these processes at the cellular level in rainbow trout acclimated to 10°C. Changes in [Ca^2+^]_i_ are directly related to changes in force of contraction (Yue, 1987) and, thus, Δ[Ca^2+^]_i_ is often used an index of force in isolated cardiomyocytes. Using this approach, the first objective of this study was to characterise how [Ca^2+^]_i_ in ventricular cardiomyocytes is affected by adrenergic stimulation and increasing pacing frequencies, either at 10°C or after an acute temperature increase to 22°C (see Table 1 for study objectives and hypotheses). We hypothesised that Δ[Ca^2+^]_i_ would decrease as pacing frequency increases and with acute warming, mirroring declines in peak tension in rainbow trout muscle-strip preparations (Hove-Madsen, 1992; Shiels and Farrell, 1997; Shiels et al., 1998). Next, we hypothesised that adrenaline would be a more potent inotropic agent at lower pacing frequencies and at 10°C, paralleling findings from muscle-strip studies (Aho and Vornanen, 2001; Shiels and Farrell, 1997; Shiels et al., 1998). Our second objective was to test the role of the SR in the frequency response to acute warming. Thus, our third hypothesis was that SR inhibition would reduce the magnitude of Δ[Ca^2+^]_i_ with greater effect at faster pacing frequencies and with acute warming (Shiels and Farrell, 1997; Shiels et al., 1998). Our fourth, and final, hypothesis was that warming would allow fish cardiomyocytes to produce Δ[Ca^2+^]_i_ at higher frequencies, but would induce a greater number of alternans (assessed as irregularly shaped Δ[Ca^2+^]_i_), and that this would be compounded by SR inhibition and adrenergic stimulation.

**Table 1.** Summary table of the objectives and hypotheses of the present study

Objective	Hypothesis
Characterise how adrenaline and pacing frequency alter [Ca^2+^]_i_, either at 10°C or 22°C (acute warming): hypotheses 1 and 2	(1) Increasing pacing frequency and acute warming will attenuate Ca^2+^ transients (Δ[Ca^2+^]_i_)
	(2) Adrenaline will have a greater inotropic effect at 10°C and at lower pacing frequencies
Characterise SR function as frequency increases, either at 10°C or 22°C: hypotheses 3 and 4	(3) Sarcoplasmic reticulum (SR) inhibition will attenuate Δ[Ca^2+^]_i_ more greatly at higher pacing frequencies and acute warming
	(4) Cardiomyocytes will produce normal Δ[Ca^2+^]_i_ at higher frequencies during acute warming than at 10°C; however, SR inhibition will limit this ability

List of symbols and abbreviations

AD: adrenaline
Ca^2+^: calcium ion
[Ca^2+^]_i_: intracellular (free cytosolic) Ca^2+^ concentration
Δ[Ca^2+^]_i_: intracellular Ca^2+^ transient
EC coupling: excitation–contraction coupling
GLMM: generalised linear mixed-effects model
*K*_d_
: dissociation constant
RyR: ryanodine receptor
SERCA: sarcoplasmic/endoplasmic reticulum Ca^2+^-ATPase
SR: sarcoplasmic reticulum

## MATERIALS AND METHODS

### Experimental animals

Adult female rainbow trout [*Oncorhynchus mykiss* (Walbaum 1792); *n*=16, weighing between 140 and 298 g, average of 219.1±10.0 g; Table S1] were purchased from Dunsop Bridge Trout Farm, Ltd (Clitheroe, Lancashire, UK), and transported to the University of Manchester (Manchester, UK). Fish were then placed in 500-litre tanks with recirculating dechlorinated water, maintained at 10°C and held under a 12 h:12 h light:dark cycle. Fish were fed three times a week to satiation with commercial fish pellets. Water quality (water temperature, chlorine, ammonia, nitrite and nitrate) was checked every second day, and tank water was changed three times per week. Fish husbandry and experimental procedures were in accordance with local animal-handling protocols and adhered to the United Kingdom Home Office legislation. Fish were acclimated to their holding tanks for at least 2 weeks before experimentation. Fish were euthanised by a percussive blow to the head (to stun and kill the fish), followed by spinal severing and brain pithing.

### Cardiomyocyte isolation

Ventricular cardiomyocytes were isolated by enzymatic dissociation, as previously described (Vornanen, 1998). Briefly, the heart was rapidly excised and cannulated by retrograde perfusion through the bulbous arteriosus into the ventricle. The heart was initially perfused with isolation solution for 8 to 10 min to remove blood, debris and Ca^2+^ from the chambers and spongy tissue, and then with enzymatic dissociation solution for 5 to 7 min (see Table 2 for all solution recipes). After perfusion, the atrium, bulbous arteriosus and sinus venosus were removed and the ventricle was minced into small pieces (≤3 mm). Individual ventricular cardiomyocytes were then released by gentle agitation, using a Pasteur pipette. Finally, cardiomyocytes were suspended in isolation solution in a conical tube, which was placed on ice and stored at 4°C for up to 5 h. A total of 17 individual adult trout were used in this study, with heart masses between 193.0 and 610.6 mg (377.5±25.8 mg; see Table S1 for a complete list of morphometrics).

**Table 2.** Compositions of solutions used in the present study

Component	Isolation solution	Dissociation solution^§^	Perfusion saline
NaCl (mmol l^−1^)	100	100	150
KCl (mmol l^−1^)	10	10	5.4
NaH_2_PO_4_ (mmol l^−1^)	–	–	0.4
KH_2_PO_4_ (mmol l^−1^)	1.2	1.2	–
MgSO_4_ (mmol l^−1^)	4	4	–
CaCl_2_ (mmol l^−1^)	–	–	2
HEPES (mmol l^−1^)	10	10	10
Taurine (mmol l^−1^)	50	50	–
Glucose (mmol l^−1^)	20	20	10
Trypsin type IX-S (mg ml^−1^)	–	0.5	–
Collagenase type IA (mg ml^−1^)	–	0.75	–
BSA (mg ml^–1^)	–	0.75	–
pH	6.9*	6.9*	7.7^‡^

pH adjusted with *KOH or ^‡^NaOH at room temperature (20°C). ^§^Enzymes were dissolved in the solution, just prior to enzymatic perfusion. Collagenase type IA (cat. no. C9891) and trypsin type IX-S (cat. no. T0303) were purchased from Sigma-Aldrich.

### Loading of cardiomyocytes with fluorescent indicator and blockers

[Ca^2+^]_i_ was measured by loading resuspended cardiomyocytes with the acetoxymethyl (AM)-ester cell-permeant fluorescent indicator Fura-2 AM (0.075 μmol l^−1^; Invitrogen, Loughborough, UK) for 10 min at room temperature (22°C). To prevent indicator leakage across the plasma membrane, the Fura-2 was de-esterified by resuspending cardiomyocytes in fresh isolation solution for 15 to 20 min. Cardiomyocytes were then randomly assigned to one of four treatment groups: (1) control, (2) adrenaline (1 μmol l^−1^), (3) SR inhibition (ryanodine+thapsigargin) and (4) adrenaline+SR inhibition. Cardiomyocytes in the SR inhibition groups were incubated with ryanodine (10 μmol l^−1^; a RyR blocker) and thapsigargin (2 μmol l^−1^; a SERCA blocker), to inhibit SR Ca^2+^ cycling, for 30 min at room temperature before experiments commenced. Ryanodine and thapsigargin were dissolved in dimethyl sulfoxide (DMSO) and administered with a final dose of 0.1% DMSO during the 30-min incubation; control and adrenaline-treated cells were incubated with 0.1% DMSO, without inhibitors. The concentrations of ryanodine and thapsigargin used in this study have been shown to be effective in inhibiting Ca^2+^ cycling by the SR in rainbow trout (Shiels and White, 2005). The adrenaline dose of 1 μmol l^−1^ represents a circulating level of catecholamine in stressed rainbow trout (Gerwick et al., 1999; Nakano and Tomlinson, 1967) and produces a 2.3-fold increase in the peak current density of *I*_Ca_ in rainbow trout ventricular cardiomyocytes (Vornanen, 1998). Although high, this is a submaximal dose and falls within the dose–response curve of myocardial tension development, as a function of adrenaline, in rainbow trout acclimated to either 8°C or 18°C (Keen et al., 1993). Cardiomyocytes were not pre-treated with adrenaline, but exposed to adrenaline via the perfusion saline during the experiment. Each cardiomyocyte was tested under one of these conditions only, and, when possible, cardiomyocytes from each heart were used for all treatments.

### Experimental protocol

Following the loading protocol, cardiomyocytes were placed in a flow-through perfusion bath (model Series RC-21BRFS, Warner Instruments, Hamden, CT, USA), which was equipped with field-stimulation electrodes that were connected to a stimulator (model SD-9, Grass Instruments, Astro-Med, Inc., West Warwick, RI, USA). Cardiomyocytes were bathed in trout perfusion solution (Table 2) and stimulated with ascending pacing frequencies (from 0.2 to 2 Hz) that span the physiologically relevant range for the temperatures tested (Shiels and Farrell, 1997; Wood et al., 1979). To test the cardiomyocytes at their acclimation temperature of 10°C, the perfusion saline was chilled by thermoelectric cooling, using an In-line Heater/Cooler Peltier (model SC-20, Warner Instruments), connected to a bipolar temperature controller (CL-100, Warner Instruments). Vulcanised rubber tubing was used to insulate the perfusion lines to shuttle the saline from the Peltier to the perfusion bath.

A temperature of 22°C was chosen as the acute-warming temperature because it is within the experimental upper thermal tolerance of rainbow trout (Black, 1953), promotes SR Ca^2+^ involvement in EC coupling (Shiels et al., 1998) and represents a natural, extreme thermal stressor to rainbow trout, considering they inhabit aquatic ecosystems (stretching from southern California to Alaska) with mean maximum daily water temperatures of 18°C (US Environmental Protection Agency, 2003). A given cardiomyocyte (belonging to one of the four treatment groups provided above) was examined at either 10°C or 22°C.

### Measurement of [Ca^2+^]_i_

The perfusion bath was mounted onto an inverted, epifluorescent microscope (model Eclipse TE-2000U, Nikon, Surrey, UK) coupled to an Optoscan photomultiplier tube, monochromator and high-intensity xenon arc lamp (Cairn Research Instruments, Faversham, UK). Signals were digitised with a Digidata 1440A and analysed with pClamp 10 software (Axon Instruments, Sunnyvale, CA, USA).

All excitation light was filtered with a Nikon T510lpxru dichroic long-pass filter (Chroma Technology, Olching, Germany) and emitted light was collected using an HQ535/50m emission filter (Chroma). Fura-2 was excited by alternating wavelengths of 340 nm and 380 nm, and emission was collected at 515 nm, with input and exit slit widths each set to 10 nm. The collected 340 nm/380 nm ratiometric data were converted to [Ca^2+^]_i_ using Eqn 1 (Lattanzio, 1990):
(1)

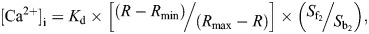
where *K*_d_ is the dissociation constant (a measure of binding affinity; *K*_d_ values were derived from Shiels et al., 2002a); *R* is the measured 340 nm/380 nm ratio of Fura-2 fluorescence within a cardiomyocyte; *R*_min_ and *R*_max_ are the fluorescence ratios of the calcium-free and fully calcium-bound Fura-2, respectively; and *S*_f_2__ and *S*_b_2__ are the 380 nm fluorescence values of calcium-free and calcium-bound Fura-2, respectively. The composition of our perfusion saline matched that of Shiels et al. (2002a), allowing us to use the *K*_d_ values of 383 nmol l^−1^ at 10°C and 332 nmol l^−1^ at 22°C.

### Calculations and statistical analysis

GraphPad Prism (v10, GraphPad Software, Boston, MA, USA) and SPSS (v29, IBM, Armonk, NY, USA) were used for figure production and statistical analyses, respectively. Data were tested for equal variances and normality. Generalised linear mixed-effects models (GLMMs), followed by Šidák *post hoc* tests (for multiple comparisons), were used to determine significant differences for the repeated-measures frequency trials. A GLMM allows for a comprehensive statistical analysis when there are multiple sources of random variability. In our study, the GLMMs considered the variability between and within cardiomyocyte treatment groups, as a single measure of residual variance cannot account for both. Graphed data were plotted using either quadratic (*Y*=*B*_0_+*B*_1_*X*+*B*_2_*X*^2^) or straight-line (*Y*=*mX*+*B*) equations to produce best-fit curves, with 95% confidence intervals (CIs).

To analyse differences in Ca^2+^ transients (Δ[Ca^2+^]_i_), diastolic and systolic [Ca^2+^]_i_, Δ[Ca^2+^]_i_ capacity, time to peak and time to half-decay, the fixed factors were temperature, drug treatment (adrenaline and SR inhibition) and pacing frequency, and the random factor was the individual cardiomyocytes. Because the data for all variables were not normally distributed, we transformed the data using either log or square-root functions and fitted them to normal or gamma distributions, depending on which model provided the best fit (Bolker, 2015), as indicated by Akaike's information criterion (AIC).

To analyse the occurrence of detectable, rhythmic Δ[Ca^2+^]_i_ in cardiomyocytes, we fitted data to Poisson distributions, as cardiomyocytes would produce either normal Δ[Ca^2+^]_i_ or irregularly shaped Δ[Ca^2+^]_i_ (i.e. alternans) in response to temperature, pacing frequency and drug treatment. For this analysis, the fixed factors were temperature, drug treatment (adrenaline and SR inhibition) and pacing frequency, and the random factor was the individual cardiomyocytes. The data relating to this analysis were also transformed using either log or square-root functions and fitted to normal or gamma distributions, depending on AIC value.

Δ[Ca^2+^]_i_ was calculated by subtracting the diastolic [Ca^2+^]_i_ value from the systolic [Ca^2+^]_i_ value, and the Δ[Ca^2+^]_i_ capacity was calculated by multiplying individual Δ[Ca^2+^]_i_ values by the pacing frequency at which they were measured. Ca^2+^ alternans were defined as Δ[Ca^2+^]_i_ that were irregularly shaped, skipped or alternated between large and small amplitudes (i.e. inconsistent shapes, sizes and durations). *Q*_10_ effects were calculated using Eqn 2:
(2)

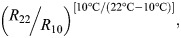
where *R*_22_ is the mean treatment value at 22°C and *R*_10_ is the mean treatment value at 10°C. *Q*_10_ effects were analysed with Student's *t*-tests, as the data were normally distributed. All data are presented as either means±s.e.m. or as fitted curves ±95% CIs (see Table S2) and considered significantly different when *P*≤0.05.

## RESULTS

### Effect of frequency and adrenaline on Δ[Ca^2+^]_i_ at 10°C (acclimation temperature)

**Fig. 1.**
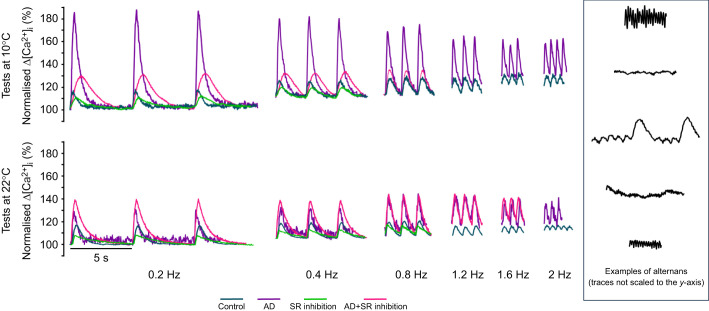
**Representative traces of normalised Ca^2+^ transients (Δ[Ca^2+^]_i_) from rainbow trout ventricular cardiomyocytes.** Cardiomyocytes were isolated from fish acclimated to 10°C, then randomly assigned to one of four drug-treatment groups and subjected to increasing pacing frequencies (0.2 to 2.0 Hz) at test temperatures of either 10°C (top panel) or 22°C (bottom panel). The treatment groups were control (cyan traces), adrenaline (AD; purple traces), sarcoplasmic reticulum (SR) inhibition (green traces) and AD combined with SR inhibition (pink traces). Ryanodine and thapsigargin were used to inhibit SR Ca^2+^ cycling. Δ[Ca^2+^]_i_ was normalised to the baseline Δ[Ca^2+^]_i_ value at 0.2 Hz and plotted as a percentage change from the baseline. Note that individual cells were exposed to one treatment and one test temperature only. An Δ[Ca^2+^]_i_ alternan is characterised by irregular shapes, sizes and durations, as illustrated by the waveforms in the right-most panel (black traces).

**Fig. 2.**
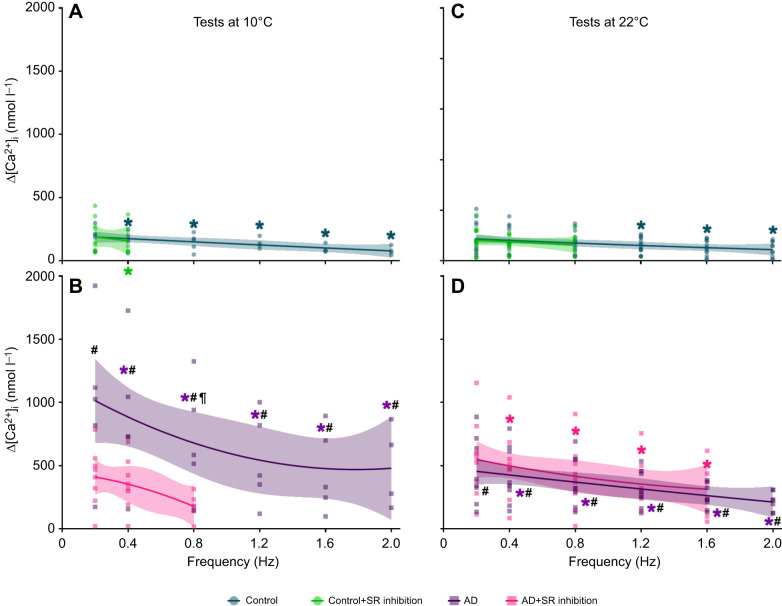
**Effects of acute warming, pharmacological intervention and pacing frequency on Ca^2+^ transients (Δ[Ca^2+^]_i_) of rainbow trout ventricular cardiomyocytes.** Cardiomyocytes were isolated from fish acclimated to 10°C, then randomly assigned to one of four drug-treatment groups and subjected to increasing pacing frequencies (0.2 to 2.0 Hz) at test temperatures of either 10°C (A,B) or 22°C (C,D). The treatment groups were control (cyan circles and traces), AD (purple squares and traces), SR inhibition (green circles and traces) and AD+SR inhibition (pink squares and traces). Ryanodine and thapsigargin were used to inhibit SR Ca^2+^ cycling. Statistical significance was revealed by repeated-measures, generalised linear mixed-effects models, followed by Šidák *post hoc* tests (for multiple comparisons). These are indicated by different symbols for frequency-dependent effects (* indicates a significant difference from 0.2 Hz) and drug-dependent effects (#, control versus AD; ¶, AD versus AD+SR inhibition). Quadratic and straight-line equations were used to produce best-fit curves, with 95% confidence intervals. Values are significant when *P*≤0.05. Initial *n*-values for tests at 10°C=5 control, 5 AD, 10 SR inhibition and 8 AD+SR inhibition cardiomyocytes, from *N*=3, 3, 4 and 3 fish, respectively. Initial *n*-values for tests at 22°C=14 control, 8 AD, 11 SR inhibition and 9 AD+SR inhibition cardiomyocytes, from *N*=6, 4, 5 and 4 fish, respectively. Note that *n*-values decreased with faster pacing frequencies, due to cell death, arrhythmogenesis or alternans, as shown in Table 5.

**Fig. 3.**
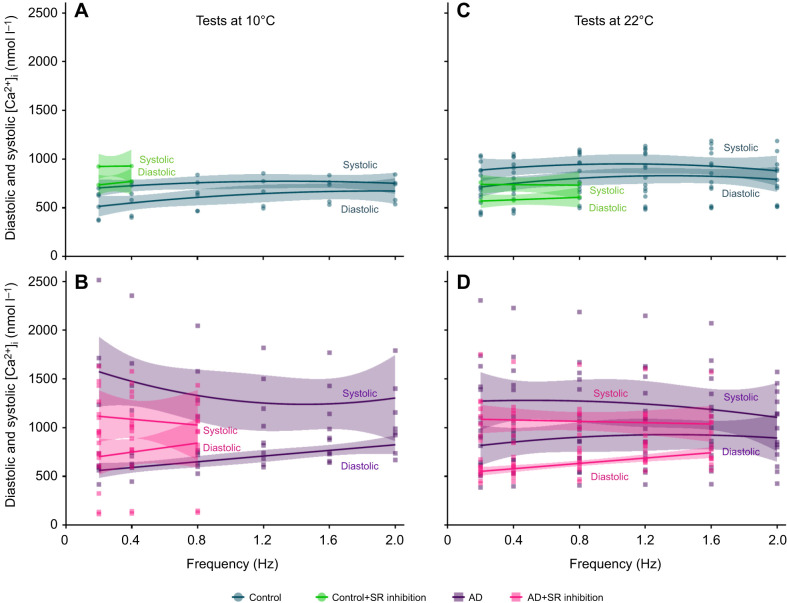
**Effects of acute warming, pharmacological intervention and pacing frequency on intracellular Ca^2+^ concentrations ([Ca^2+^]_i_) of rainbow trout ventricular cardiomyocytes.** Cardiomyocytes were isolated from fish acclimated to 10°C, then randomly assigned to one of four drug-treatment groups and subjected to increasing pacing frequencies (0.2 to 2.0 Hz) at test temperatures of either 10°C (A,B) or 22°C (C,D). The treatment groups were control (cyan circles and traces), AD (purple squares and traces), SR inhibition (green circles and traces) and AD+SR inhibition (pink squares and traces). Ryanodine and thapsigargin were used to inhibit SR Ca^2+^ cycling. Statistically significant values are presented in Table 3. Diastolic and systolic [Ca^2+^]_i_ values are represented by lower and upper traces that have the same symbol and colour, respectively. Quadratic and straight-line equations were used to produce best-fit curves, with 95% confidence intervals. The *n*-values for tests at 10°C and 22°C are the same as described in Fig. 2.

Under control conditions, there were significant, gradual decreases in Δ[Ca^2+^]_i_ as frequency was increased from 0.4 Hz onwards (Fig. 1 upper panel, Fig. 2A). The frequency-dependent changes in Δ[Ca^2+^]_i_ were driven by significant elevations in diastolic [Ca^2+^]_i_, whereas systolic [Ca^2+^]_i_ increased slightly, albeit significantly (Fig. 3A, Table 3).

**Table 3.** Diastolic and systolic intracellular Ca^2+^ concentrations ([Ca^2+^]_i_) of rainbow trout ventricular cardiomyocytes acclimated to 10°C and tested at 10°C or 22°C

Variable	Test temperature	Drug treatment	Pacing frequency					
			0.2 Hz	0.4 Hz	0.8 Hz	1.2 Hz	1.6 Hz	2.0 Hz
Diastolic [Ca^2+^]_i_ (nmol l^−1^)	10°C	Control	505.6±57.9	561.1±68.5*	612.8±69.0*	633.5±82.8*	666.5±71.5*	675.3±71.1*
		AD	555.4±35.6	596.7±42.6*	650.2±42.4*	711.4±51.0*	758.7±53.7*	829.1±75.8*
		SR inhibition	735.0±47.6^‡^	770.8±64.7*^‡^	–	–	–	–
		AD+SR inhibition	731.5±98.6	775±107.5*	830.4±152.0*	–	–	–
	22°C	Control	713.6±56.1^¥^	747.2±56.7*^¥^	813.9±60.8*^¥^	813.3±65.5*^¥^	834.9±74.5*^¥^	787.4±75.6*
		AD	828.9±123.9	849.6±122*^€^	878.1±127.0*^€^	921.7±137.9*^€^	963.2±145.2*	870.9±119.6*
		SR inhibition	572.7±39.6	575.0±43.7*^‡^	610.3±58.4*^‡^	–	–	–
		AD+SR inhibition	548.5±25.6	576.9±25.8*	633.9±31.1*	701.1±34.2*	729.9±54.7*	–
Systolic [Ca^2+^]_i_ (nmol l^−1^)	10°C	Control	700.7±43.5	729.1±56.3	759.5±55.6*	772.7±71.1*	761.6±62.8*	755.0±59.2*
		AD	1566.9±293.8^#^	1473.5±267.5^#^	1352.4±219.3^#^	1254.2±187.5*^#^	1212.7±178.4*^#^	1323.3±181.4*^#^
		SR inhibition	924.5±59.9^‡^	929.2±81.1*^‡^	–	–	–	–
		AD+SR inhibition	1142.0±163.6	1128.6±158.5	1007.9±188.9	–	–	–
	22°C	Control	886.4±76.6^¥^	910.2±74.0^¥^	959.1±73.9^¥^	933.7±74.3^¥^	936.3±82.1^¥^	878.7±82.8^¥^
		AD	1282.8±208.5	1273.6±197.1	1256.4±191.6	1236.1±185.1	1218.0±176	1087.9±144.3
		SR inhibition	760.2±57^$^	702.0±55.9^$^	749.0±77.4	–	–	–
		AD+SR inhibition	1107.0±111.3	1064.6±103.7*	1039.9±106.4*	1077.2±88.1*	1032.2±133.1*	–

Cardiomyocytes were isolated from fish acclimated to 10°C, then randomly assigned to one of four drug-treatment groups and subjected to increasing pacing frequencies (0.2 to 2.0 Hz) at a test temperature of either 10°C or 22°C. The drug-treatment groups were control, adrenaline (AD), inhibited sarcoplasmic reticulum (SR) Ca^2+^ cycling and AD combined with inhibited SR Ca^2+^ cycling. Ryanodine and thapsigargin were used to inhibit SR Ca^2+^ cycling. Statistical significance was revealed by repeated-measures, generalised linear mixed-effects models, followed by Šidák *post hoc* tests; for frequency-dependent effects, * denote a significant difference from the 0.2 Hz value (one-tailed tests); for temperature-dependent effects (10°C versus 22°C; two-tailed tests), significant differences are denoted by ¥ (control) and € (AD); and for drug-dependent effects (two-tailed tests), by # (control versus AD) and ‡ (control versus SR inhibition). Values are means±s.e.m. and significant when *P*≤0.05. The *n*-values for tests at 10°C and 22°C are the same as described in Fig. 2.

**Fig. 4.**
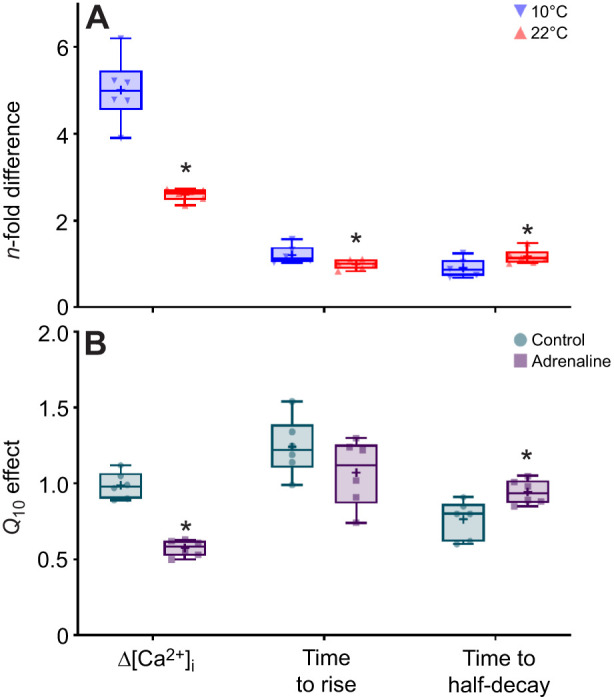
***n*****-fold differences and** ***Q*****_10_ effects of the mean values of intracellular Ca**^**2+**^ **transients (Δ[Ca**^**2+**^**]**_**i**_**) and their rise and half-decay times in trout ventricular cardiomyocytes.**Cardiomyocytes were isolated from fish acclimated to 10°C, then randomly assigned to either the control or adrenaline treatment groups and subjected to increasing pacing frequencies (0.2 to 2.0 Hz) at a test temperature of either 10°C or 22°C. (A) The effect of adrenaline, at either 10°C or 22°C, is described by *n*-fold differences, which were calculated as the quotient of the mean Δ[Ca^2+^]_i_ values of the adrenaline group divided by the control group, for each respective frequency and test temperature. (B) The effect of temperature, in either the control or adrenaline-treated group, is described by *Q*_10_ values, which were calculated using Eqn 2 for each respective frequency and treatment. Asterisks (*) denote temperature-dependent significant differences in *n*-fold differences or adrenaline-dependent significant differences in *Q*_10_ values. Each box extends from the 25th to the 75th percentiles and displays a median value (horizontal line), a mean value (+), individual data points (*n*=6 for each box), and a vertical line to indicate minimum and maximum values.

Adrenaline had a significant, positive inotropic effect on Δ[Ca^2+^]_i_ across all pacing frequencies (Fig. 1 upper panel, Fig. 2B), with elevations in Δ[Ca^2+^]_i_ ranging from 3.90- to 6.20-fold, compared with control cardiomyocytes (Fig. 4). Yet, the effect of adrenaline on systolic and diastolic [Ca^2+^]_i_ varied and was frequency dependent; at low pacing frequencies, adrenaline substantially elevated Δ[Ca^2+^]_i_ by increasing both diastolic and systolic [Ca^2+^]_i_, but as pacing frequency increased, diastolic [Ca^2+^]_i_ progressively rose, whereas systolic [Ca^2+^]_i_ gradually decreased, before levelling off at a steady state (Fig. 3B, Table 3). Furthermore, adrenaline only had a significant inotropic effect on systolic [Ca^2+^]_i_, relative to control cells, but not on diastolic [Ca^2+^]_i_ (Table 3).

These results suggest that the positive inotropic effects of adrenaline are diminished at high frequencies, as evidenced by the convergence of Δ[Ca^2+^]_i_ values in the control and adrenaline groups at the highest pacing frequencies (Fig. 2A,B). This was also observed in the Δ[Ca^2+^]_i_ kinetics, as there were only modest (albeit significant) differences in the times to rise (at 0.2 Hz only) and half-decay at 0.4, 0.8 and 2 Hz (Table 4).

**Table 4.** Effects of acute warming, pharmacological intervention and pacing frequency on the times to rise and half-decay of the Ca^2+^ transients (Δ[Ca^2+^]_i_) of rainbow trout ventricular cardiomyocytes

Variable	Test temperature	Drug treatment	Pacing frequency					
			0.2 Hz	0.4 Hz	0.8 Hz	1.2 Hz	1.6 Hz	2.0 Hz
Time to rise (ms)	10°C	Control	212.0±30.0	177.3±21.1	159.6±15.6*	125.8±13.7*	110.2±11.9*	92.3±6.1*
		AD	333.4±39.7	236.5±30.1*	186.6±18.7*	133.3±10.9*	112.6±13.2*	98.9±13.2*
		SR inhibition	433.8±63.4	263.3±30.3*^‡^	–	–	–	–
		AD+SR inhibition	691.7±56.6^¶^	542.2±60.9*^¶^	334.9±39.5*^¶^	–	–	–
	22°C	Control	208.4±25.2	206.7±25.2	196.7±27*	165.0±19.7*	156.6±15.8*	143.1±16.3*^¥^
		AD	236.8±30.1	216.7±33.1*	201.4±31.8*	195.8±32.3*	151.7±22.8*	137.7±20.3*
		SR inhibition	187.1±23.9^$^	151.3±15.8*^$^	119.8±14.9*	–	–	–
		AD+SR inhibition	291.0±18.6^£^	232.0±17.5*^£^	188.0±16*^£^	159.2±12.6*	118.9±20.7*	–
Time to half-decay (ms)	10°C	Control	492.3±83.1	443.4±22.6	258.4±7.0*	213.0±14.2*	152.4±11.6*	103.9±8.7*
		AD	365.5±21	302.0±25.4*^#^	219.4±11.8*^#^	187.6±15.4*	159.1±10.1*	129.7±3.7*^#^
		SR inhibition	535.1±106	388.1±40.5	–	–	–	–
		AD+SR inhibition	716.6±58.8^¶^	503.0±43.2*^¶^	325±29.8*^¶^	–	–	–
	22°C	Control	267.7±28.2^¥^	252.0±22.7^¥^	196.5±17.1*^¥^	162.1±11.1*^¥^	125.3±11.2*	92.8±12.7*
		AD	291.6±31.1^€^	258.8±32.2*^€^	222.7±16.5*^€^	165.3±9.0*^€^	158.8±16.0*^€^	140.7±16.1*^€^
		SR inhibition	515.9±71.4^‡^	320±30.5*	278.2±20.5*^‡^	–	–	–
		AD+SR inhibition	570.4±51.8^£¶^	396.5±30.2*^£¶^	234.6±16.9*^£^	194.9±12.8*^¶^	135.6±15.3*	–

Cardiomyocytes were isolated from fish acclimated to 10°C, then randomly assigned to one of four drug-treatment groups and subjected to increasing pacing frequencies (0.2 to 2.0 Hz), at test temperatures of either 10°C (left panels) or 22°C (right panels). The treatment groups were control, AD, SR inhibition and AD+SR inhibition. Ryanodine and thapsigargin were used to inhibit SR Ca^2+^ cycling. Statistical significance was revealed by repeated-measures, mixed-effects generalised linear mixed-effects models, followed by Šidák *post hoc* tests; for frequency-dependent effects, * denote a significant difference from the 0.2 Hz value (one-tailed tests); for temperature-dependent effects (10°C versus 22°C; two-tailed tests), significant differences are denoted by ¥ (control), € (AD), $ (SR inhibition) and £ (AD+SR inhibition); and for drug-dependent effects (one-tailed tests), by # (control versus AD), ‡ (control versus SR inhibition) and ¶ (AD versus AD+SR inhibition). Values are means±s.e.m. and significant when *P*≤0.05. The *n*-values for tests at 10°C and 22°C are the same as described in Fig. 2.

### Effect of inhibiting SR Ca^2+^ cycling at 10°C

Inhibition of SR Ca^2+^ cycling (with ryanodine and thapsigargin) did not affect the amplitude of Δ[Ca^2+^]_i_ relative to the control group, but severely impeded maximal pacing frequency, with Δ[Ca^2+^]_i_ becoming irregular at frequencies above 0.4 Hz (Fig. 1 upper panel, Fig. 2A). Although Δ[Ca^2+^]_i_ was unaffected by SR inhibition, the time to rise of Δ[Ca^2+^]_i_ was significantly slowed relative to the control group, whereas the time to half-decay was unaffected (Table 4).

**Fig. 5.**
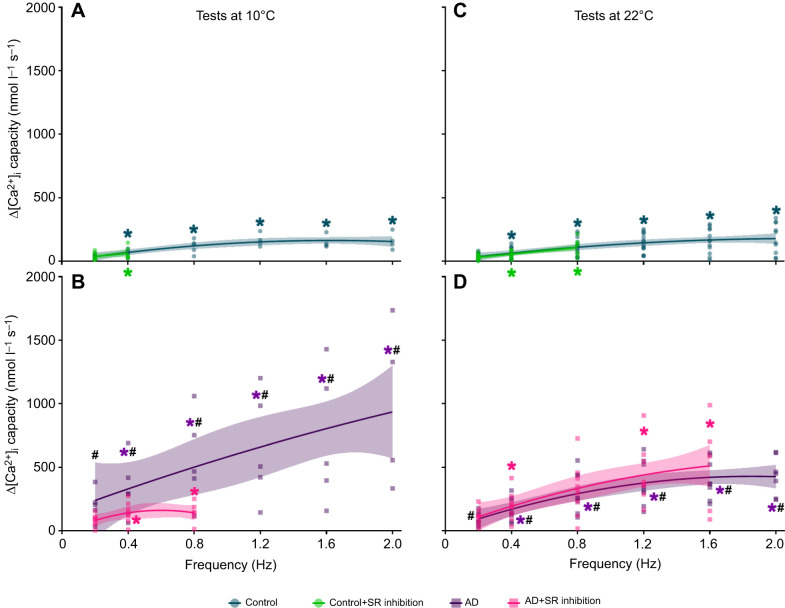
**Effects of acute warming, pharmacological intervention and pacing frequency on the Ca^2+^-transient (Δ[Ca^2+^]_i_) capacities of rainbow trout ventricular cardiomyocytes.** Cardiomyocytes were isolated from fish acclimated to 10°C, then randomly assigned to one of four drug-treatment groups and subjected to increasing pacing frequencies (0.2 to 2.0 Hz) at test temperatures of either 10°C (A,B) or 22°C (C,D). The treatment groups were control (cyan circles and traces), AD (purple squares and traces), SR inhibition (green circles and traces) and AD+SR inhibition (pink squares and traces). Ryanodine and thapsigargin were used to inhibit SR Ca^2+^ cycling. Statistical significance was revealed by repeated-measures, generalised linear mixed-effects models, followed by Šidák *post hoc* tests (for multiple comparisons). These are indicated by different symbols for frequency-dependent (* indicates a significant difference from 0.2 Hz), temperature-dependent and drug-dependent (#, control versus AD) effects. Quadratic and straight-line equations were used to produce best-fit curves, with 95% confidence intervals. Values are significant when *P*≤0.05. The *n*-values for tests at 10°C and 22°C are the same as described in Fig. 2.

SR inhibition tended to reduce Δ[Ca^2+^]_i_ in adrenaline-treated cardiomyocytes, but this was significantly lower only at 0.8 Hz (Fig. 2B). Nevertheless, adrenergic stimulation enabled SR-inhibited cardiomyocytes to produce Δ[Ca^2+^]_i_ at frequencies up to 0.8 Hz (Fig. 1 upper panel, Fig. 2B). The combination of adrenaline and SR inhibition had no significant effect on the Δ[Ca^2+^]_i_ capacity relative to adrenaline treatment alone (Fig. 5B), but significantly slowed the Ca^2+^-cycling kinetics (Table 4).

### Effect of frequency and adrenaline on Δ[Ca^2+^]_i_ at 22°C (acute warming)

Under control conditions, there was a significant, gradual and frequency-dependent decrease in Δ[Ca^2+^]_i_ (Fig. 1 lower panel, Fig. 2C). The changes in Δ[Ca^2+^]_i_ were caused by a significant rise in diastolic [Ca^2+^]_i_, whereas systolic [Ca^2+^]_i_ was unaltered (Fig. 3C, Table 3).

Adrenaline elicited a significant, positive inotropic effect on Δ[Ca^2+^]_i_ across all pacing frequencies relative to the control group (Fig. 1 lower panel, Fig. 2D). The frequency-dependent reduction in Δ[Ca^2+^]_i_ with adrenaline treatment was due to a significant, gradual rise in diastolic [Ca^2+^]_i_, whereas systolic [Ca^2+^]_i_ remained unchanged (Fig. 3D, Table 3). Moreover, the *n*-fold change in Δ[Ca^2+^]_i_ was steady across pacing frequencies (2.35- to 2.74-fold differences at 22°C, compared with 3.90- to 6.20-fold differences at 10°C; Fig. 4).

### Effect of inhibiting SR Ca^2+^ cycling at 22°C

Inhibition of SR Ca^2+^ cycling (with ryanodine and thapsigargin) had no significant effect on Δ[Ca^2+^]_i_ (Fig. 1 lower panel, Fig. 2C). Moreover, acute warming allowed SR-inhibited cardiomyocytes to pace at faster frequencies, with Δ[Ca^2+^]_i_ alternans tending to occur after 0.8 Hz, compared with 0.4 Hz at 10°C.

When adrenaline was administered to SR-inhibited cardiomyocytes, they were able to pace at higher frequencies, and Δ[Ca^2+^]_i_ only became irregular at frequencies higher than 1.6 Hz, compared with 0.8 Hz in SR-inhibited cardiomyocytes alone. Nevertheless, the combination of adrenaline and SR inhibition did not affect Δ[Ca^2+^]_i_ (Fig. 1 lower panel, Fig. 2D), even though there was a significant slowing of Ca^2+^-cycling kinetics (Table 4). These results suggest that the mobilisation of SR Ca^2+^ is not required to maintain Δ[Ca^2+^]_i_, when pacing frequencies reach upper physiologically relevant frequencies in ventricular cardiomyocytes from 10°C-acclimated trout acutely warmed to 22°C.

### Effect of acute warming: differences between control cardiomyocytes

Under control conditions, acute warming had no significant effect on Δ[Ca^2+^]_i_ (Fig. 2A,C), as diastolic and systolic [Ca^2+^]_i_ were similar between cold (acclimation temperature) and acutely warmed cardiomyocytes, across all pacing frequencies (Fig. 3A,C, Table 3). However, acute warming significantly depressed Ca^2+^-cycling kinetics in a frequency-dependent manner; the time to rise was significantly slower at 2 Hz, whereas the time to half-decay was significantly faster between 0.2 and 1.2 Hz (Table 4).

Adrenaline had no significant effect on Δ[Ca^2+^]_i_ (Fig. 2B,D). Similar to control conditions at 10°C, the frequency-dependent reduction in Δ[Ca^2+^]_i_ was caused by rises in diastolic [Ca^2+^]_i_, although these differences did not reach levels of statistical significance (Fig. 3B,D, Table 3). However, when comparing data from 22°C versus 10°C, the *n*-fold changes in Δ[Ca^2+^]_i_ and rise time were significantly lower and in half-decay time were significantly higher (Fig. 4). Moreover, there were significant differences in the *Q*_10_ effects of Δ[Ca^2+^]_i_ and the time to half-decay between control and adrenaline-treated cardiomyocytes (Fig. 4). For Δ[Ca^2+^]_i_, the control *Q*_10_ effect was significantly higher than the adrenaline *Q*_10_ effect (Fig. 4). For the time to half-decay, the control *Q*_10_ effect was significantly lower than the adrenaline *Q*_10_ effect (Fig. 4). These results demonstrate the limited capacity of adrenaline to increase Δ[Ca^2+^]_i_ at acutely warm temperatures, while also altering the Ca^2+^-cycling kinetics.

### Effect of acute warming: inhibition of SR Ca^2+^ cycling

Acute warming, in combination with inhibited SR Ca^2+^ cycling (with ryanodine and thapsigargin), had no effect on Δ[Ca^2+^]_i_ compared with the same group at 10°C (Figs 2A,C and 3A,C, Table 3), despite significantly slowing the time to rise (Table 4). Adrenaline combined with the SR inhibitors also had no effect on Δ[Ca^2+^]_i_ (Fig. 2B,D), despite slowing of the time to rise and time to half-decay (Table 4).

### Onset of irregular Ca^2+^ transients

The development of irregular Δ[Ca^2+^]_i_, or alternans (i.e. inconsistent shapes, sizes and durations; see Fig. 1), was assessed across all treatments and at each test temperature. At 10°C, most cardiomyocytes failed to either produce regular Δ[Ca^2+^]_i_ or contract at frequencies higher than 2 Hz (control and adrenaline groups), 0.4 Hz (SR-inhibited group) and 0.8 Hz (adrenaline+SR-inhibited group) (Table 5). At 22°C, most cardiomyocytes failed to either produce regular Δ[Ca^2+^]_i_ or contract at frequencies higher than 2 Hz (control and adrenaline groups), 0.8 Hz (SR-inhibited group) and 1.6 Hz (adrenaline+SR-inhibited group) (Table 5).

**Table 5.** The percentage of pacing cardiomyocytes with detectable rhythmic Ca^2+^ transients

Test temperature	Treatment	Initial *n*-value	Pacing frequency					
			0.2 Hz	0.4 Hz	0.8 Hz	1.2 Hz	1.6 Hz	2.0 Hz
10°C	Control	5	100%	100%	100%	80%	80%	80%
	AD	5	100%	100%	100%	100%	100%	80%
	SR inhibition	10	100%	60%	–	–	–	–
	AD+SR inhibition	8	100%	87.5%	62.5%	–	–	–
22°C	Control	14	100%	100%	100%	100%	85.7%	71.4%
	AD	8	100%	100%	100%	100%	100%	87.5%
	SR inhibition	11	100%	81.8%	54.5%	–	–	–
	AD+SR inhibition	9	100%	100%	100%	88.9%	55.6%	–

Cardiomyocytes were isolated from fish acclimated to 10°C, then randomly assigned to one of four drug-treatment groups and subjected to increasing pacing frequencies (0.2 to 2.0 Hz) at a test temperature of either 10°C or 22°C. The drug-treatment groups were control, AD, SR inhibition and AD+SR inhibition. Ryanodine and thapsigargin were used to inhibit SR Ca^2+^ cycling. The *n*-values for tests at 10°C and 22°C are the same as described in Fig. 2.

### Test statistics

The repeated-measures GLMM revealed significant main effects of, and interactions between, temperature, pharmacological intervention (adrenaline and/or SR inhibition) and frequency (Table S3). Therefore, adrenaline and acute warming mitigated the effects of irregular Δ[Ca^2+^]_i_ as pacing frequency increased.

## DISCUSSION

**Fig. 6.**
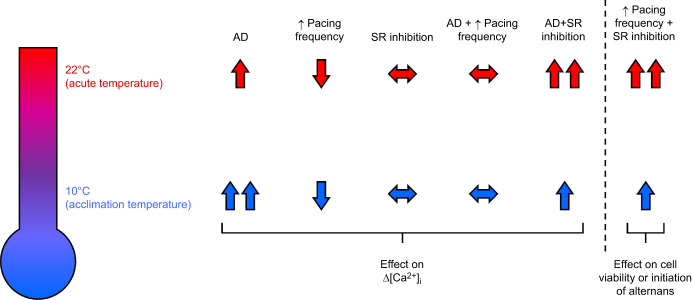
**Summary figure of the interplay between acute warming, increasing pacing frequency and pharmacological intervention.** The intracellular Ca^2+^ transients (Δ[Ca^2+^]_i_) and viability (including the development of alternans) of isolated cardiomyocytes of rainbow trout acclimated to 10°C are affected by either the singular or combined effects of acute warming (22°C), adrenergic stimulation with AD, inhibited Ca^2+^ cycling by the SR and increasing pacing frequency (from 0.2 to 2.0 Hz). Blue and red arrows represent changes to Δ[Ca^2+^]_i_ and viability during tests at the acclimation temperature (10°C) and with acute warming (22°C), respectively. Arrows pointing up (↑) and down (↓) indicate significantly increased and decreased changes in a variable, respectively; double arrows (↑↑ or ↓↓) indicate significant, temperature-dependent differences between 10°C and 22°C; and horizontal, two-way arrows (↔) indicate no significant changes.

Warming is a stressor many fish face as they traverse thermoclines, and it is the most potent environmental modulator of cardiac function in piscine species (Farrell et al., 2009). Its influence on the fish heart depends on the severity and duration of the temperature increase (Shiels et al., 2024). Durations can be measured from days and seasons (chronic) to minutes and hours (acute), the latter of which was investigated in this study, by measuring the physiological effect acute warming has on the ventricular cardiomyocytes of rainbow trout subjected to increasing pacing frequencies, adrenergic stimulation and inhibition of SR Ca^2+^ cycling. To understand the interplay between these key regulators of fish heart function at the cellular level (which we summarise in Fig. 6), we characterised frequency-induced changes to Δ[Ca^2+^]_i_, at either the acclimation temperature of the fish (10°C) or following acute warming (22°C). In measuring [Ca^2+^]_i_, we hoped to reveal how diastolic and systolic [Ca^2+^]_i_ are affected by regulators of cardiac function, and how these regulators impact Δ[Ca^2+^]_i_ and the development of alternans.

Although our results are derived from laboratory experiments on isolated cells (i.e. they do not recapitulate either the diverse, natural ecosystems in which rainbow trout live or the whole-body responses regulating *in vivo* heart function), we were able to control environmental and experimental variables and tease apart the effects of thermal stress, adrenergic stimulation, pacing frequency and impaired SR Ca^2+^ cycling on the intrinsic physiology of cardiomyocytes. Importantly, our results align with field-based (e.g. Eliason et al., 2013; Gilbert et al., 2020), whole-animal (e.g. Ekström et al., 2014; Gilbert et al., 2019; Strowbridge et al., 2025) and tissue-based (e.g. Ekström et al., 2019, 2016; Shiels and Farrell, 1997; Shiels et al., 1998, 2002b, 2003) studies of the salmonid heart.

We found that under control conditions, Δ[Ca^2+^]_i_ in acutely warmed cardiomyocytes did not differ from cardiomyocytes at 10°C, across all pacing frequencies. Thus, we must reject our first hypothesis (Table 1). The positive inotropic effect of adrenaline was significant across all pacing frequencies in cardiomyocytes tested at 10°C and 22°C, but less potent at both higher pacing frequencies and at warm temperature, in support of our second hypothesis (Table 1). SR inhibition did not reduce the magnitude of Δ[Ca^2+^]_i_, in rejection of our third hypothesis (Table 1), and prevented cardiomyocytes from producing Δ[Ca^2+^]_i_ across the full range of tested pacing frequencies, in partial support of our fourth hypothesis (Table 1), given that acutely warmed SR-inhibited cells paced at higher frequencies than their 10°C counterparts. Finally, we reveal that acute warming increased the occurrence of irregular Δ[Ca^2+^]_i_ in control cardiomyocytes, but decreased their development in adrenaline-treated cardiomyocytes, due to significant interactions between warming, adrenaline and pacing frequency (Table 5). Under control conditions, 10 out of 14 cardiomyocytes (71.4%) exhibited irregular Δ[Ca^2+^]_i_ with acute warming at 2 Hz, compared with 4 out of 5 cardiomyocytes (80%) at 10°C (Table 5), whereas adrenergic stimulation allowed 4 out of 5 cardiomyocytes (80%) to produce normal Δ[Ca^2+^]_i_ at 10°C and 2 Hz, compared with 7 out of 8 cardiomyocytes (87.5%) at 22°C.

### Thermal plasticity of intrinsic cardiomyocyte function

Several studies on teleost fish show that acute temperature changes can alter the intrinsic physiology of the heart (e.g. Anelli et al., 2004; Farrell et al., 1996; Keen and Farrell, 1994; Overgaard et al., 2004; Rocha et al., 2007; Shiels and Farrell, 1997; Shiels et al., 1999; Tittu and Vornanen, 2001; Vornanen, 1989). Here, we showed that acute warming did not alter the magnitude of the Δ[Ca^2+^]_i_, in rejection of our first hypothesis (Table 1). Under control conditions, acutely warmed cardiomyocytes had similarly sized Δ[Ca^2+^]_i_ as cardiomyocytes tested at the colder acclimation temperature (Fig. 2A,C), whereas faster pacing frequencies caused concomitant reductions in Δ[Ca^2+^]_i_.

In fish, the activities of Ca^2+^ currents, channels and transporters (i.e. *I*_Ca_, NCX, RyR and SERCA) are highly susceptible to temperature fluctuations, which can alter cardiomyocyte function (Aho and Vornanen, 1998; Galli et al., 2011; Hove-Madsen et al., 2001; Korajoki and Vornanen, 2012, 2013; Landeira-Fernandez et al., 2004; Llach et al., 2001; Shiels et al., 2006; Shiels et al., 2000, 2002b). However, and rather surprisingly, we show that acute warming does not appreciably affect the Ca^2+^-cycling kinetics of rainbow trout ventricular cardiomyocytes, as there are only modest, albeit significant, elevations in the time to rise at the highest pacing frequency (2 Hz), and decreases in the time to half-decay at low-to-intermediate frequencies (0.2 to 2 Hz) (Table 5). The slowing of Ca^2+^-cycling kinetics suggests increased Ca^2+^ uptake by SERCA and temperature-mediated changes in myofilament Ca^2+^ sensitivity. Although we did not measure myofilament Ca^2+^ sensitivity, experimental warming can sensitise cardiac myofilaments in rainbow trout to Ca^2+^ (Gillis et al., 2000). Conversely, increased uptake by SERCA can be seen in Fig. 2A,C, in which acutely warmed SR-inhibited cardiomyocytes can contract at faster frequencies that those tested at 10°C (explained in greater detail below). It is possible that by increasing SERCA activity, acutely warmed cardiomyocytes can mobilise more Ca^2+^ from the SR to maintain the same Δ[Ca^2+^]_i_ magnitude as cardiomyocytes at 10°C. To make it so, SERCA would require a wide temperature tolerance, which is, indeed, observed in fish. Acute-warming studies have demonstrated that SERCA maintains high enzymatic activity between 5°C and 30°C in rainbow trout and coho salmon (*Oncorhynchus kisutch*) (Da Silva et al., 2011; Landeira-Fernandez et al., 2012), and between 3°C and 23°C in brown trout (*Salmo trutta*) (Vornanen, 2021), before reaching Arrhenius break points. In acutely warmed brown trout, the temperature tolerance of SERCA also keeps up with, and even surpasses, that of ventricular beats (Vornanen, 2021), showing the importance of recruiting SR Ca^2+^ when fish are subjected to a thermal stressor.

### Plasticity of the adrenergic response

Adrenaline is a powerful regulator of cardiac function. In rainbow trout, it alters inotropy and chronotropy in temperature- and frequency-dependent manners (Keen et al., 1993; Shiels and Farrell, 1997; Shiels et al., 2003). Adrenaline can recruit Ca^2+^ from the SR to enhance or protect fish cardiomyocytes from arrhythmogenesis (Cros et al., 2014). However, acute warming can desensitise fish cardiac tissues to adrenaline, either by reducing peak force production or having no effect on maximal pacing frequency (Kubly and Stecyk, 2019; Methling et al., 2012; Shiels et al., 1998, 2003), both of which reduce the heart's ability to deliver O_2_ during elevated metabolic activity. Here, we show that adrenaline has a large inotropic effect on Δ[Ca^2+^]_i_, which is tempered with increasing pacing frequency and is more robust at 10°C (Figs 2B,D and 4).

The reductions in Δ[Ca^2+^]_i_ with increasing frequency mirrors adrenaline-meditated decreases in peak tension of rainbow trout muscle strips (Aho and Vornanen, 2001; Shiels and Farrell, 1997; Shiels et al., 1998). One possible mechanism to explain the reduced potency of adrenergic stimulation at higher frequencies is the limited lusitropic response of fish cardiac tissue (i.e. the rate of relaxation after contraction). In the present study, we observed slower half-decay times at 22°C with adrenaline than at 10°C (Tables 2 and 3). Although this result appears counterintuitive, it might be related to the truncated cardiac troponin I (TnI) of fish (relative to mammalian TnI), resulting in the absence of the phosphorylation site that enhances the off-rate of cross-bridge cycling (Alderman et al., 2012; Joyce et al., 2023; Kirkpatrick et al., 2011; Solaro et al., 1976), which would slow relaxation. Considering that acute warming desensitises fish cardiac tissue to adrenaline (Shiels and Farrell, 1997; Shiels et al., 2003) and that adrenaline reduces myofilament Ca^2+^ sensitivity (Chung et al., 2016), a slower half-decay time might partly explain why the potency of adrenaline is diminished when trout cardiomyocytes are acutely warmed, resulting in impeded cardiomyocyte relaxation (i.e. reduced lusitropy), as observed in the present study.

### Thermal plasticity of arrhythmogenesis

An aim of this study was to determine whether the attenuation of the adrenergic response is frequency-dependent, SR-dependent, or whether there was an interaction between the two. Inhibition of the SR prevented cold-tested cardiomyocytes from producing normal Δ[Ca^2+^]_i_ past 0.8 Hz, even with adrenaline (Fig. 2A). These results show that the convergence of Δ[Ca^2+^]_i_ between control and adrenaline-treated cardiomyocytes is caused by impaired SR Ca^2+^ cycling. Moreover, SR inhibition does not affect the positive inotropy of adrenaline at any pacing frequency, which seems to be more pronounced at colder temperature (Shiels and Farrell, 1997; Shiels et al., 1998). We cannot determine from the present study whether adrenaline loses potency at these contraction frequencies, either because total Ca^2+^ cycling cannot sustain contractile force (a frequency-dependent effect) or because of poor SR Ca^2+^ cycling (an SR-dependent effect).

Although trans-sarcolemmal Ca^2+^ flux provides sufficient Ca^2+^ for EC coupling in most fish, the inability of adrenaline to stimulate even more Ca^2+^ release by the SR (Bovo et al., 2013; Cros et al., 2014) diminishes the positive inotropy of adrenaline on Δ[Ca^2+^]_i_ (Fig. 2B). These results contrast with observations in isolated rainbow trout cardiac muscle strips, in which SR inhibition significantly weakened force development, but did not prevent contractility (Shiels and Farrell, 1997; Shiels et al., 1998). Here, SR-inhibited cardiomyocytes either failed or became arrhythmic past pacing frequencies of 0.8 Hz and were characterised by slower Ca^2+^-cycling kinetics, even with adrenaline (Fig. 1, Table 4). Given that fish hearts require SR Ca^2+^ during stressful activity (Bovo et al., 2013; Cros et al., 2014) and for stronger force development (Anelli et al., 2004; Hove-Madsen, 1992; Keen et al., 1992, 1994; Rocha et al., 2007; Shiels and Farrell, 1997, 2000; Tittu and Vornanen, 2002), our results suggest that routine EC coupling of rainbow trout cardiomyocytes requires a combination of trans-sarcolemmal and SR Ca^2+^ fluxes at higher physiological pacing frequencies.

Acute warming likely stimulated SR Ca^2+^ cycling, as observed in other fish studies (Driedzic and Gesser, 1994; Shiels et al., 2002a; Vornanen et al., 2002). By enhancing Ca^2+^ cycling via the SR, acute warming enabled control cardiomyocytes to produce similarly sized Δ[Ca^2+^]_i_ as their 10°C counterparts. Acute warming also enabled SR-inhibited cardiomyocytes to contract up to 0.8 Hz – double the pacing rate at 10°C – before failing or become arrhythmic (Fig. 2C,D). Additionally, the combination of adrenaline and SR inhibition permitted shortening up to 1.6 Hz, which suggests a stimulatory effect on trans-sarcolemmal Ca^2+^ influx. These findings show that acutely warmed trout cardiomyocytes do not require SR Ca^2+^ stores until pacing frequencies match or exceed physiologically relevant rates.

### Perspectives

Ectothermic vertebrates have a remarkable ability to rapidly adjust physiological function in response to changes in ambient temperature. In fish, variations in temperature are most conspicuous in cardiac function (Farrell et al., 2009), and numerous studies have demonstrated the degree of thermal plasticity of the heart. This current research enterprise tackled the physiology of isolated rainbow trout ventricular cardiomyocytes and showed that acute warming alters the cellular response to adrenergic stimulation, inhibition of SR Ca^2+^ cycling and elevations in pacing frequency. We show that acute warming limits the positive inotropy of adrenaline on [Ca^2+^]_i_ across physiologically relevant pacing frequencies, in contrast to the typical response, in which adrenaline ameliorates temperature-induced increases in heart rate (which would weaken force development). Consequently, our results suggest that the intrinsic physiology of cardiomyocytes of some fish species might not be able to counteract the effects of acute warming without other compensatory mechanisms (e.g. autonomic regulation) that would enable them to better survive in the wild. Alternatively, more frequent exposure to acute temperature change might induce adaptive mechanisms (Seebacher et al., 2015), enabling better thermal tolerance of cardiac function in the future.

## Supplementary Material

10.1242/jexbio.251460_sup1 Supplementary information

Dataset 1.
