# Supplementary material for: Thermal plasticity of adrenaline-mediated, frequency-dependent calcium homeostasis in rainbow trout ventricular cardiomyocytes
Source: J Exp Biol. 2026 Feb 2;229(2):jeb251460. doi: 10.1242/jeb.251460 (PMC12912269; doi:10.1242/jeb.251460)
Supplement: Supplementary information [file jexbio-229-251460-s1.pdf]

Formulas for calculating the Ca^{2+} transients ($\Delta[Ca^{2+}]_i$) and $\Delta[Ca^{2+}]_i$ capacity are found in the Materials and Methods section in the main body of the manuscript.

Table S1. Body and heart masses of fish used in the present study. Values in bottom-most row are means ± SEM.

Body mass (g)	Heart mass (mg)
232	409.8
184	410.2
192	328
166	404.2
298	610.6
174	304.5
232	305.1
140	193
196	265.1
248	420.4
250	452.4
230	321.4
246	487
238	388.4
232	Not weighed
248	363
**219.1 ± 10.0**	**377.5 ± 25.8**

Table S2. Measured variables in ventricular cardiomyocytes of rainbow trout acclimated to 10°C and tested at 10°C (N = 5-10) and at 22C (N = 5-14). Cardiomyocytes were isolated from fish acclimated to 10°C, then randomly assigned to one of four drug-treatment groups and subjected to increasing pacing frequencies (0.2 to 2.0 Hz), at a test temperature of either 10°C or 22°C. The drug-treatment groups were control, adrenaline, inhibited sarcoplasmic reticulum Ca^{2+} cycling, and adrenaline combined with inhibited SR Ca^{2+} cycling. Ryanodine and thapsigargin were used to inhibit SR Ca^{2+} cycling. Values are means ± SEM.

**Tests at 10°C**							
		**Pacing frequency (Hz)**					
**Variable**	**Treatment**	**0.2**	**0.4**	**0.8**	**1.2**	**1.6**	**2.0**
$\Delta[Ca^{2+}]_i$ (nM)	Control	195.1 ± 36.2	168.1 ± 31.7	146.7 ± 30.1	139.2 ± 21.5	95.2 ± 16.2	79.7 ± 16.7
	Adrenaline	1011.6 ± 281.2	876.8 ± 256.2	702.2 ± 200.4	542.8 ± 160.8	454 ± 147.9	494.2 ± 163.8
	SR inhibition	189.5 ± 41.7	158.4 ± 49.1	-	-	-	-
	Adrenaline + SR inhibition	410.6 ± 81.3	353.6 ± 71.9	177.5 ± 40.3	-	-	-
Diastolic $[Ca^{2+}]_i$ (nM)	Control	505.6 ± 57.9	561.1 ± 68.5	612.8 ± 69	633.5 ± 82.8	666.5 ± 71.5	675.3 ± 71.1
	Adrenaline	555.4 ± 35.6	596.7 ± 42.6	650.2 ± 42.4	711.4 ± 51	758.7 ± 53.7	829.1 ± 75.8
	SR inhibition	735 ± 47.6	770.8 ± 64.7	-	-	-	-
	Adrenaline + SR inhibition	731.5 ± 98.6	775 ± 107.5	830.4 ± 152	-	-	-
Systolic $[Ca^{2+}]_i$ (nM)	Control	700.7 ± 43.5	729.1 ± 56.3	759.5 ± 55.6	772.7 ± 71.1	761.6 ± 62.8	755 ± 59.2
	Adrenaline	1566.9 ± 293.8	1473.5 ± 267.5	1352.4 ± 219.3	1254.2 ± 187.5	1212.7 ± 178.4	1323.3 ± 181.4
	SR inhibition	924.5 ± 59.9	929.2 ± 81.1	-	-	-	-
	Adrenaline + SR inhibition	1142 ± 163.6	1128.6 ± 158.5	1007.9 ± 188.9	-	-	-
$\Delta[Ca^{2+}]_i$ capacity (nM s^{-1})	Control	39 ± 7.2	67.2 ± 12.7	117.3 ± 24.1	167 ± 25.8	152.2 ± 26	159.4 ± 33.5
	Adrenaline	202.3 ± 56.2	350.7 ± 102.5	561.7 ± 160.3	651.4 ± 192.9	726.4 ± 236.7	988.4 ± 327.6
	SR inhibition	37.9 ± 8.3	63.3 ± 19.6	-	-	-	-
	Adrenaline + SR inhibition	82.1 ± 16.3	141.4 ± 28.8	142 ± 32.2	-	-	-
Time to rise (ms)	Control	212.0 ± 30.0	177.3 ± 21.1	159.6 ± 15.6	125.8 ± 13.7	110.2 ± 11.9	92.3 ± 6.1
	Adrenaline	333.4 ± 39.7	236.5 ± 30.1	186.6 ± 18.7	133.3 ± 10.9	112.6 ± 13.2	98.9 ± 13.2
	SR inhibition	433.8 ± 63.4	263.3 ± 30.3	-	-	-	-
	Adrenaline + SR inhibition	684.5 ± 63.6	542.2 ± 60.9	334.9 ± 39.5	-	-	-
Time to half-decay (ms)	Control	492.3 ± 83.1	443.4 ± 22.6	258.4 ± 7.0	213.0 ± 14.2	152.4 ± 11.6	103.9 ± 8.7
	Adrenaline	365.5 ± 21.0	302 ± 25.4	219.4 ± 11.8	187.6 ± 15.4	159.1 ± 10.1	129.7 ± 3.7
	SR inhibition	535.1 ± 106.0	388.1 ± 40.5	-	-	-	-
	Adrenaline + SR inhibition	713.8 ± 66.6	503.0 ± 43.2	325.0 ± 29.8	-	-	-
**Tests at 22°C**							
		**Pacing frequency (Hz)**					
**Variable**	**Treatment**	**0.2**	**0.4**	**0.8**	**1.2**	**1.6**	**2.0**
$\Delta[Ca^{2+}]_i$	Control	172.9 ± 32.1	163 ± 25.7	145.2 ± 24	120.4 ± 16.4	101.4 ± 17.6	91.3 ± 19

(nM)	Adrenaline	453.9 ± 93.6	423.9 ± 82.7	378.3 ± 70.1	314.4 ± 51.9	254.8 ± 34.2	217 ± 28.7
	SR inhibition	175.8 ± 26.9	126.9 ± 21.4	138.7 ± 31.6	-	-	-
	Adrenaline + SR inhibition	558.6 ± 100.8	487.8 ± 91.2	406 ± 85.3	376.2 ± 67.6	302.4 ± 87.4	-
Diastolic $[Ca^{2+}]_i$ (nM)	Control	713.6 ± 56.1	747.2 ± 56.7	813.9 ± 60.8	813.3 ± 65.5	834.9 ± 74.5	787.4 ± 75.6
	Adrenaline	828.9 ± 123.9	849.6 ± 122	878.1 ± 127	921.7 ± 137.9	963.2 ± 145.2	870.9 ± 119.6
	SR inhibition	572.7 ± 39.6	575 ± 43.7	610.3 ± 58.4	-	-	-
	Adrenaline + SR inhibition	548.5 ± 25.6	576.9 ± 25.8	633.9 ± 31.1	701.1 ± 34.2	729.9 ± 54.7	-
Systolic $[Ca^{2+}]_i$ (nM)	Control	886.4 ± 76.6	910.2 ± 74	959.1 ± 73.9	933.7 ± 74.3	936.3 ± 82.1	878.7 ± 82.8
	Adrenaline	1282.8 ± 208.5	1273.6 ± 197.1	1256.4 ± 191.6	1236.1 ± 185.1	1218 ± 176	1087.9 ± 144.3
	SR inhibition	760.2 ± 57	702 ± 55.9	749 ± 77.4	-	-	-
	Adrenaline + SR inhibition	1107 ± 111.3	1064.6 ± 103.7	1039.9 ± 106.4	1077.2 ± 88.1	1032.2 ± 133.1	-
$\Delta[Ca^{2+}]_i$ capacity (nM s^{-1})	Control	34.6 ± 6.4	65.2 ± 10.3	116.2 ± 19.2	144.4 ± 19.7	162.2 ± 28.2	182.6 ± 37.9
	Adrenaline	90.8 ± 18.7	169.6 ± 33.1	302.7 ± 56.1	377.3 ± 62.3	407.7 ± 54.7	434.1 ± 57.3
	SR inhibition	35.2 ± 5.4	50.8 ± 8.5	111 ± 25.3	-	-	-
	Adrenaline + SR inhibition	111.7 ± 20.2	195.1 ± 36.5	324.8 ± 68.2	451.4 ± 81.2	504.8 ± 169.4	-
Time to rise (ms)	Control	208.4 ± 25.2	206.7 ± 25.2	196.7 ± 27.0	165.0 ± 19.7	156.6 ± 15.8	143.1 ± 16.3
	Adrenaline	236.8 ± 30.1	216.7 ± 33.1	201.4 ± 31.8	195.8 ± 32.3	151.7 ± 22.8	137.7 ± 20.3
	SR inhibition	187.1 ± 23.9	151.3 ± 15.8	119.8 ± 14.9	-	-	-
	Adrenaline + SR inhibition	291.0 ± 18.6	232 ± 17.5	188.0 ± 16.0	159.2 ± 12.6	118.9 ± 20.7	-
Time to half-decay (ms)	Control	267.7 ± 28.2	252 ± 22.7	196.5 ± 17.1	162.1 ± 11.1	125.3 ± 11.2	92.8 ± 12.7
	Adrenaline	291.6 ± 31.1	258.8 ± 32.2	222.7 ± 16.5	165.3 ± 9.0	158.8 ± 16.0	140.7 ± 16.1
	SR inhibition	515.9 ± 71.4	320.0 ± 30.5	278.2 ± 20.5	-	-	-
	Adrenaline + SR inhibition	570.4 ± 51.8	396.5 ± 30.2	234.6 ± 16.9	194.9 ± 12.8	135.6 ± 15.3	-

Table S3. Test statistics of the main fixed effects of various aspects of rainbow trout ventricular cardiomyocyte physiology. Cardiomyocytes were isolated from fish acclimated to 10°C, then randomly assigned to one of four drug-treatment groups and subjected to increasing pacing frequencies (0.2 to 2.0 Hz), at a test temperature of either 10°C or 22°C. The drug-treatment groups were control, adrenaline, inhibited sarcoplasmic reticulum Ca^{2+} cycling, and adrenaline combined with inhibited SR Ca^{2+} cycling. Ryanodine and thapsigargin were used to inhibit SR Ca^{2+} cycling. Repeated-measures, generalised linear mixed-effects models were used to reveal the effects of each fixed factor: temperature, drug treatment, and pacing frequency.

Variable	Factor	F-value	df1	df2	P-value
$\Delta[Ca^{2+}]_i$	Temperature	0.29	1	261	0.589
	Drug treatment	12.95	3	261	≤0.001
	Frequency	37.65	5	261	≤0.001
	Temperature * Drug treatment	0.62	3	261	0.605
	Temperature * Frequency	1.93	5	261	0.089
	Drug treatment * Frequency	2.43	11	261	0.007
	Temperature * Drug treatment * Frequency	0.60	8	261	0.782
$\Delta[Ca^{2+}]_i$ capacity	Temperature	0.32	1	260	0.572
	Drug treatment	13.17	3	260	≤0.001
	Frequency	288.93	5	260	≤0.001
	Temperature * Drug treatment	0.66	3	260	0.579
	Temperature * Frequency	1.18	5	260	0.319
	Drug treatment * Frequency	1.99	11	260	0.030
	Temperature * Drug treatment * Frequency	1.16	8	260	0.324
Diastolic $[Ca^{2+}]_i$	Temperature	0.02	1	260	0.885
	Drug treatment	0.62	3	260	0.604
	Frequency	60.66	5	260	≤0.001
	Temperature * Drug treatment	6.78	3	260	≤0.001
	Temperature * Frequency	8.25	5	260	≤0.001
	Drug treatment * Frequency	1.69	11	260	0.076
	Temperature * Drug treatment * Frequency	3.17	8	260	0.002
Systolic $[Ca^{2+}]_i$	Temperature	0.14	1	260	0.711
	Drug treatment	5.06	3	260	0.002
	Frequency	6.83	5	260	≤0.001
	Temperature * Drug treatment	2.80	3	260	0.040
	Temperature * Frequency	5.18	5	260	≤0.001
	Drug treatment * Frequency	9.99	11	260	≤0.001
	Temperature * Drug treatment * Frequency	3.11	8	260	0.002
Time to rise	Temperature	9.21	1	253	0.003
	Drug treatment	13.69	3	253	≤0.001
	Frequency	61.90	5	253	≤0.001
	Temperature * Drug treatment	5.82	3	253	≤0.001
	Temperature * Frequency	6.47	5	253	≤0.001
	Drug treatment * Frequency	3.73	11	253	≤0.001
	Temperature * Drug treatment * Frequency	1.73	8	253	0.093
Time to half-decay	Temperature	6.55	1	253	0.011
	Drug treatment	13.38	3	253	≤0.001
	Frequency	96.56	5	253	≤0.001
	Temperature * Drug treatment	1.84	3	253	0.141
	Temperature * Frequency	2.44	5	253	0.035
	Drug treatment * Frequency	4.55	11	253	≤0.001

	Temperature * Drug treatment * Frequency	1.97	8	253	0.051

			Student's t-test statistics		
**Analysis**	**Variable**	**Comparison**	**t-value**	**df**	**P-value**
n-fold differences	$\Delta[Ca^{2+}]_i$	10°C vs. 22°C	9.85	10	≤0.001
	Time to rise		2.21	10	0.050
	Time to half-decay		-2.35	10	0.040
Q_{10} effects	$\Delta[Ca^{2+}]_i$	Control vs. adrenaline	9.78	10	≤0.001
	Time to rise		1.43	10	0.184
	Time to half-decay		-2.99	10	0.014

Dataset 1.

Available for download at
https://journals.biologists.com/jeb/article-lookup/doi/10.1242/jeb.251460#supplementary-data
